# The Receding Specialty of Infectious Diseases and Implications for U.S. Healthcare

**DOI:** 10.1093/ofid/ofaf756

**Published:** 2025-12-10

**Authors:** Gonzalo Bearman, Rebecca Mullin, Priya Nori

**Affiliations:** Department of Medicine, Virginia Commonwealth University, Richmond, Virginia, USA; Department of Medicine, Virginia Commonwealth University, Richmond, Virginia, USA; Division of Infectious Diseases, Department of Medicine, Albert Einstein College of Medicine, Montefiore Health System, Bronx, New York, USA

**Keywords:** antibiotic stewardship, infection prevention, infectious diseases, pandemic threats, public healthrecedingrisk

## Abstract

Infectious diseases physicians serve on the frontline of disease prevention, outbreak mitigation, and direct patient care across the healthcare continuum. To reverse troubling trends in the ID workforce, policymakers, healthcare administrators, and the public must understand the essential societal role of ID physicians. Solutions include adopting new compensation models, loan forgiveness for medical specialties where demand exceeds supply, reformed immigration policies for staffing medically underserved areas and reinvestment in public health. A receding ID workforce will result in delayed ID specialty care, worse clinical outcomes, worsening antibiotic resistance, increased healthcare costs, decreased pandemic preparedness, and an overall sicker nation.

Infectious diseases account for approximately 5–15% of deaths in the United States annually [1]. From 2020 to 2022, COVID-19 was the number three cause of mortality, with 1 in 10 Americans dying of the disease [2]. Despite the increasing pervasiveness of infections, the risk of new pandemic threats and resurging vaccine-preventable illnesses, infectious diseases (ID) physicians are a receding segment of the U.S. physician workforce. While ID salaries have historically been low, recent troubling trends such as political antagonism, and defunding of infectious disease priorities may further disincentivize the ID workforce [3]. As a specialty, ID's reach spans beyond direct patient care-and drives healthcare quality through antibiotic stewardship, infection prevention, and public health efforts connecting acute care, long-term, and ambulatory care. Therefore, ID is unique in its expansive scope, and a contraction in workforce threatens the safety of individual patients and entire communities. While these facts are well known to ID, it is incumbent on us to warn hospital administrators and policy makers of what is at stake for individual patients and healthcare infrastructure if ID further recedes as a specialty.

## EVIDENCE OF A WORKFORCE IN DECLINE

There are approximately 14 000 ID physicians in the U.S, < 1% of the physician workforce [4]. The impacts of this shortage are disproportionate. Eighty percent of U.S. counties have no ID doctors, and another 10% have very few. There were 6424 physicians with active ID licenses in 2008 and 9136 by 2018, but only 9774 in 2025 [5]. Nearly 50% of ID training programs now go unfilled, further contracting the pipeline [6]. Meanwhile, infectious disease threats grow due to climate change, a declining vaccine uptake, and an increasingly obese, aging and ailing population with diabetes, immune-suppressing therapies, cancer, organ transplants, and implantable medical devices.

## CLINICAL AND PUBLIC HEALTH VALUE OF ID SPECIALISTS

Infectious diseases consultation results in shorter hospital lengths of stay, reduced cost, and improved outcomes for complex infections [7–9]. ID physicians staff hospitals and clinics but also serve as public health experts at the Centers for Disease Control and Prevention (CDC) and regional public health departments, where they conduct novel research, develop molecular diagnostics,and respond to multi-state outbreaks. The CDC coordinates with the World Health Organization on surveillance for both domestic and international threats like avian- influenza, viral hemorrhagic fevers, *Candida auris*, and others [10, 11], and both organizations rely on ID-trained physician guidance. Most antibiotic overuse fueling drug-resistant bacteria occurs outside hospitals, in ambulatory settings, and in non-human health sectors. ID specialists at the CDC and WHO coordinate surveillance and stewardship across all settings [12].

Without access to local ID expertise, other specialties must rely on public health guidance to manage threats like measles, influenza and COVID-19. Unfortunately, public health guidelines like those for age-appropriate vaccines are now fraught with political interference, leaving regional consortia of policymakers and public health experts to step in to preserve access and insurance coverage [13].

## DRIVERS OF ID WORKFORCE DECLINE

**Figure 1.**
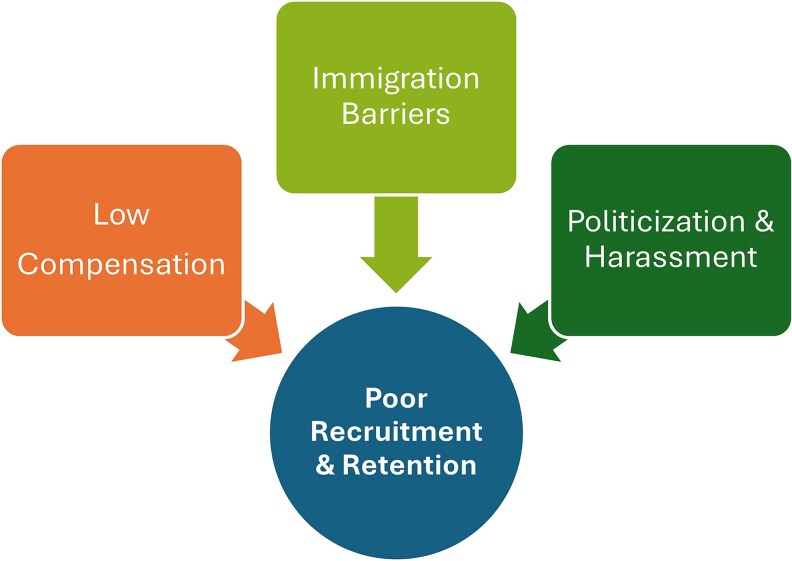
Drivers of ID Workforce Decline.

Interconnected structural barriers undermine the recruitment and retention of ID physicians (Figure 1).

## COMPENSATION

ID is among the lowest-paid specialties, with salaries significantly below those of general internists and hospitalists, despite requiring two to three additional years of training [14]. Furthermore, recent proposed Medicare reimbursement cuts for hospital-based physicians, like ID, will further exacerbate this remuneration disparity (Health Resources & Services Administration). Additionally, ID physicians spend on average [15] 8.4 hours of time in the EHR for every 8 hours of patient care, more than most specialists. Excessive EHR burden is [16] associated with higher burnout, and when combined with additional years of training and comparatively lower salaries, disincentivizes candidates.

More competitive salary models exist [17]. Hospitalist salaries are not exclusively driven by productivity measured in Relative Value Units (RVUs), and instead reflect an increasing demand for their services and their impact on cost, decreased length of stay, and improvement in quality metrics (eg, HCAHPS scores) [18, 19]. Hospitalists often rely on subsidies from hospitals to support their salaries, as provider fee billing does not adequately cover costs [18, 19]. This is justified by their overall service and value provided (eg, increased hospital throughput).

## IMMIGRATION BARRIERS

International medical graduates (IMGs) played a critical role in meeting U.S. healthcare workforce needs through the Immigration and Nationality Act of 1965, which facilitated entry from countries like India and the Philippines to address shortages. Current immigration pathways are cumbersome and unpredictable, preventing qualified international physicians from filling critical workforce gaps. Furthermore, a September 2025 executive order requiring $100 000 for new H1B visa applications further exacerbated the strain on these physicians and their employers. While physicians on the H1B visa pathway are reportedly exempt [20, 21], international physicians legally employed within our healthcare system remain vulnerable to erratic enforcement of immigration policies by the current administration.

## POLITICIZATION AND HARASSMENT

An escalation in threats to the public health workforce further disincentivizes ID as a career. Targeting of healthcare and public health workers escalated during COVID and in its aftermath. Harassment is cited as a major factor leading to departure from the public health workforce [22, 23]. In August 2025, a gunman opened fire at the Atlanta CDC campus, a violent and unprecedented act precipitated by increasingly politicized rhetoric about public health [24–26]. The subsequent firing of the acting CDC director, a career public health scientist who refused to acquiesce to the dangerous anti-science policies of the HHS director, followed by the resignation in protest of 3 senior CDC officials (2 of 3 who oversaw crucial infectious disease prevention and control efforts), culminated in numerous healthcare organizations [27], HHS employes [28], and Congressional Democrats demanding his resignation [25, 26].

## IMPACT ON COLLEAGUES AND HEALTH SYSTEMS

High revenue-generating hospital services like organ transplantation, chemotherapy, joint replacements, etc. would not have successful outcomes without ID specialists creating antibiotic prophylaxis and healthcare-associated infection prevention protocols, conducting surveillance, and providing consultative care when infectious complications arise. Hospitalists especially rely on ID consults as part of an effective care model [29]. Inaccessibility of ID consultative services would result in decreased guideline-concordant care, increased LOS, less effective antibiotic management for bloodstream infections and sepsis.

Moreover, programs like Infection Prevention and Control ensure that hospitals do not lose billions in reimbursement from preventable infections [30]. Hospital administrators expect ID services at every healthcare touchpoint, from the emergency department to inpatient units, to post-acute care settings; therefore, administrators should be accountable for recruiting and retaining their ID workforce through competitive pay.

However, the pursuit of bolstering the ID workforce or championing fair compensation may be challenged by hospital investments in artificial intelligence (AI)-based automation as an alternative to hiring more ID specialists. AI, which is rapidly evolving to augment direct patient care, drug optimization and infection prevention surveillance can potentially fill ID care gaps in under-resourced areas, but liability and patient disclosure implications of AI-based clinical decisions remain unknown [31].

## REINVESTING IN THE ID WORKFORCE

In addition to the ongoing ID physician compensation advocacy by the Infectious Diseases Society of America (IDSA), there are available solutions to these issues, with precedent or successful examples from other hospital services (Table 1) [32]. While some require enduring advocacy and systemic policy change, other “stopgap” solutions can be implemented more easily.

**Table 1.** Reinvesting in the ID Workforce to Improve Recruitment and Retention

Problem	Solutions
Low relative compensation	Compensate fairly and equitably Change to a compensation structure of clinical productivity plus value-based care (ie, Hospitalist model) Fund public health and ID loan repayment legislation
Immigration barriers	Draw from past legislative precedents which increased the workforce to meet societal needs Ensure that physicians on legal work visas and are protected from intimidation and harassment
Adverse political environment	Speak out, vote, engage peacefully Law makers should stand against political rhetoric targeting public health and work to enhance the public perception of ID

First, policymakers can push for loan repayment for physicians in medically underserved areas, and for specialties with growing population demands like ID. The Bio-Preparedness Workforce Pilot Program, enacted in 2022 with bipartisan support in the House and Senate, proposes loan repayment for the public health workforce or ID specialists working with the medically underserved, but funding has not yet been appropriated [33].

Nearly 80% of US physicians are employed by hospitals and corporate entities [34]. Institutions must compensate ID specialists competitively and fairly, commensurate with hospitalist physicians. Conversely, Infectious Diseases productivity is typically measured in RVUs, tying their output to revenue from professional billing. As a non-procedural specialty, infectious diseases physicians earn relatively few RVUs for the clinical work they perform. For example, an ID physician completing a new patient consult billing CPT code 99223 may expect approximately 3.86 work RVUs for this service, while procedures such as a balloon angioplasty billing CPT 92920 earns more than double that at 9.85 [35]. This use of RVUs as a gauge of revenue associated with an ID physician drives compensation down in attempts to match revenue to expense. However, ID consultants are involved in many aspects of hospitalist patient care [36], from initial diagnosis to antibiotic management of acute infections, transitions of care, and outpatient follow-up [36]. ID's healthcare impact entails direct patient care and value-based services like infection prevention, stewardship, and outpatient antibiotic therapy (OPAT) programs. Value-based care models of compensation have been implemented effectively in other specialties; West Virginia University Medicine increased its Heart and Vascular Institute revenue by a factor of 10 after shifting from an RVU-based model to a value-based care model, while successfully increasing their workforce and patient volume [37].

Reformed immigration policies are also vital to staffing medically underserved areas. Streamlining pathways for international physicians to work in underserved areas while expediting their path to citizenship would immediately strengthen access to ID expertise. Furthermore, we should not tolerate intimidation of any physicians living and working legally in the U.S.

Re-investment in public health is essential. Stable funding for local and state health departments and the CDC ensures that there are sufficient ID physicians and epidemiologists to meet population needs. Moreover, they must be guaranteed protection from dangerous political agendas.

Acknowledging that organizational change takes years, interim measures to augment the current ID workforce are urgently needed. For instance, an appropriately resourced and reimbursed telehealth service can expand ID expertise to underserved, rural areas, and hospitals can deploy advanced practice for lower acuity ID consultative work (in-person or remotely), allowing ID physicians to focus on higher complexity consults with better reimbursement.

Additionally, ID fellowship programs with unfilled positions could create dual training tracks with popular specialties like critical care. If combined positions could be offered outside the traditional National Resident Matching Program structure, they could serve as an immediate and proactive solution to declining fellowship recruitment. Short-term, creative solutions are needed immediately but should not replace long-term, structural investments in the ID workforce by policymakers and health systems.

In summary, ID physicians serve on the frontline of disease prevention, outbreak mitigation, and direct patient care across the healthcare continuum. To reverse current troubling trends, policymakers and the public must understand the essential societal role of ID physicians. A receding ID workforce will result in delayed ID specialty care, worse clinical outcomes, worsening antibiotic resistance, increased healthcare costs, decreased pandemic preparedness, and an overall sicker nation [38].
